# Promoting Research Culture at Indus Health Network

**DOI:** 10.12669/pjms.36.ICON-Suppl.1861

**Published:** 2020-01

**Authors:** Shaukat Ali Jawaid

**Affiliations:** Chief Editor

Indus Hospital is a part of the ever expanding Indus Health Network which is providing state of the art tertiary care facilities to the people free of cost. Over the last five years this institution has made tremendous progress and also earned Charter to become one of the medical universities functioning in Pakistan. In order to promote research culture they have also established a full-fledged Research Department and have been collaborating with numerous national and international institutions. Hence when they approached us on February 2^nd^ 2019 to publish a Special Supplement of Pakistan Journal of Medical Sciences to showcase the research work of their faculty members on the occasion of their forthcoming International Conference scheduled to be held in January 2020, we decided for a meeting to discuss the issue in detail. Hence the first meeting was held on February 13^th^ 2019 followed by another meeting on March 1^st^ 2019. It was decided in principle to publish the supplement. Other decisions taken in this meeting included the following:

1. The authors of the manuscripts to be published in the Supplement will follow the instructions and guidelines for authors by Pakistan Journal of Medical Sciences both in letter and spirit. Each manuscript to be accompanied by Letter of Undertaking signed by all the listed authors and Ethics Committee approval.
2. They will follow the authorship policy of the Journal.
3. Indus Hospital will select two Guest Editors who will collect all the manuscripts, review them for their suitability, then arrange for their Peer Review by at least two experts, get the papers revised and then send it to Pakistan Journal of Medical Sciences.
4. These manuscripts will undergo another editing by the staff of Pakistan Journal of Medical Sciences before final acceptance, those not found in order will be returned to be further revised and once they are finalized, they will be uploaded on the Journal website.
5. PDF files of the manuscripts will be sent to the Guest Editors to be passed on to the respective authors for proof reading before final publication.

Later a Memorandum of Understanding giving details of other commitments on either side was signed on April 17^th^ 2019 and a Workshop on Peer Review was conducted for some of the senior faculty members, guest editors and other related staff to facilitate them in editing, peer review of the manuscripts before they are forwarded to the Journal.

The Guest Editors and other members of the team in Indus Hospital looking after this project did a commendable job which ensured that the selected manuscripts were of good quality and covered interesting topics which will help further enhance the image and credibility of Indus Health Network. However, as usually happens, there were still some problems with References, some needed further editing, some tables, figures had to be re-drawn, some manuscript being too lengthy had to be shortened and revised and all this went on for almost over six months before the manuscripts were finalized. Indus Hospital team was told to follow different deadlines to accomplish this project in time and they successfully managed that. This must also have been a great learning experience for them and helped them in their professional capacity building. Not only that, they must have realized that publishing a good quality peer review journal is a very stressful and frustrating experience. Particularly dealing with the authors is an uphill task and then coordination with the Reviewers was also not so easy. Many of these manuscripts must have been revised more than once before getting a final shape. Yet another meeting was held during the month of September 2019 to sort out some of these issues related to certain manuscripts which had to be resolved.

On our part in order to encourage the authors and as a special gesture of good will towards Indus Health Network, we had agreed to include even some of those manuscripts like Case Reports, Manuscripts based on routine surveys, lengthy Reviews and CME manuscripts which otherwise has a very low priority with us and normally we do not entertain such manuscripts. Working with the Indus Hospital Team looking after this project in general and the Guest Editors in particular has been a wonderful experience and we wish the organizers of the ICON 2020 a great success.

